# Indocyanine Green Near-Infrared Fluoroangiography Is a Useful Tool in Reducing the Risk of Anastomotic Leakage Following Left Colectomy

**DOI:** 10.3389/fsurg.2022.850256

**Published:** 2022-03-29

**Authors:** Miriam Neddermeyer, Veit Kanngießer, Elisabeth Maurer, Detlef K. Bartsch

**Affiliations:** Department of Visceral-, Thoracic- and Vascular Surgery, Philipps-University Marburg, Marburg, Germany

**Keywords:** indocyanine green near-infrared fluoroangiography, anastomotic leakage, sigmoid resection, rectal resection, colorectal surgery

## Abstract

**Purpose:**

To evaluate whether visualization of the colon perfusion with indocyanine green near-infrared fluoroangiography (ICG-NIFA) reduces the rate of anastomotic leakage (AL) after colorectal anastomosis.

**Methods:**

Patients who underwent elective left colectomy, including all procedures involving the sigmoid colon and the rectum with a colorectal or coloanal anastomosis, were retrospectively analyzed for their demographics, operative details, and the rate of AL. Univariate and multivariate analyses were used to compare patients with and without ICG-NIFA-based evaluation.

**Results:**

Overall, our study included 132 colorectal resections [70 sigmoid resections and 62 total mesorectal excisions (TMEs)], of which 70 (53%) were performed with and 62 (47%) without ICG-NIFA. Patients' characteristics were similar between both the groups. The majority of the procedures [91 (69%)] were performed by certified colorectal surgeons, while 41 (31%) operations were supervised teaching procedures. In the ICG-NIFA group, bowel perfusion could be visualized by fluorescence (dye) in all 70 cases, and no adverse effects related to the fluorescent dye were observed. Following ICG-NIFA, the transection line was changed in 9 (12.9%) cases. Overall, 10 (7.6%) patients developed AL, 1 (1.4%) in the ICG-NIFA group and 9 (14.5%) in the no-ICG-NIFA group (*p* = 0.006). The multivariate analysis revealed ICG-NIFA as an independent factor to reduce AL.

**Conclusion:**

These results suggest that ICG-NIFA might be a valuable tool to reduce the rate of AL in sigmoid and rectal resections in an educational setting.

## Introduction

Anastomotic leakage (AL) is a major complication in colorectal surgery, with rates varying between 1 and 25% depending on the type and extent of resection (1, 2). AL is often associated with high morbidity, including sepsis, the need for reoperation, and stoma creation, and sometimes even mortality. Risk factors for post-operative AL such as high body mass index (BMI), pre-operative radiochemotherapy, and low distance of the anastomosis from the anal verge are challenging to modify (3). Therefore, the surgical community has undertaken significant efforts to optimize the surgical technique to reduce the rate of AL. AL can result from technical failures such as improper use of the stapler, poor suturing, increased tension, or suboptimal blood perfusion. While technical details can be controlled and corrected easily, identifying optimal blood perfusion at the transected bowel edges remains challenging. At present, most surgeons still assess the anastomotic perfusion by the color of the bowel wall, peristalsis of the bowel, pulsation of the arteries, and bleeding at the transection surface. However, this assessment is entirely subjective depending on the surgeon's experience and might lead to misinterpretation.

In 1976, it was discovered that protein-bound indocyanine green (ICG) could emit near-infrared fluorescence (NIF) (4). In 1992, ICG was first used for angiography of the ocular fundus (5), while in 2006, ICG-NIFA was utilized to identify sentinel lymph nodes in colorectal surgery (6). Nowadays, ICG-NIFA is increasingly considered an effective tool to accurately determine the blood supply at the anastomotic site in routine surgical practice. This present trend is based on several recently published retrospective studies as well as meta-analyses that reported a potential reduction of the AL rate when using ICG-NIFA. However, most of these studies were performed by expert colorectal surgeons, and few prospective randomized trials have shown inconclusive results (7–13).

The present study aimed to evaluate the feasibility of ICG-NIFA in routine practice and its impact on AL rates after sigmoid resection and total mesorectal excision (TME) for benign and malignant diseases at a single training clinic.

## Materials and Methods

### Methods

The ICG-NIFA technique was first introduced to our hospital in May 2017. After an initial supervised introduction of the ICG-NIFA principle and system to the surgical staff, the use of ICG-NIFA turned into a method of choice for our responsible certified colorectal surgeons. Subsequently, the hospital documentation system and the certified colorectal cancer database were screened for patients who underwent colorectal resections between January 2017 and January 2020 at the Department of Visceral-, Thoracic-, and Vascular Surgery, Philipps-University, Marburg. To focus on two highly standardized procedures, only data of patients who underwent either elective sigmoid resections or total mesorectal excisions (TME) with colorectal anastomosis with or without the use of ICG-NIFA were selected. As the surgical approach neither influences the bowel perfusion nor the anastomotic healing rate, conventionally open, laparoscopic, and robotic resections were included. Exclusion criteria were age <18 years, emergency procedures, and discontinuous resections.

The primary endpoints were the feasibility of ICG-NIFA in routine practice and the rate of AL in procedures performed with or without ICG-NIFA support. Hospital stay and in-hospital mortality were considered secondary endpoints. The approval for data collection and analysis for this study was obtained from the Ethics Committee of the Marburg University hospital.

Demographic patients' data, intraoperative and post-operative data, and pathology were retrospectively collected and analyzed. The severity of post-operative complications was graded according to the Dindo-Clavien classification (14). AL is defined as “a defect of the intestinal wall at the anastomotic site leading to a communication between the intra- and extraluminal compartments. A pelvic abscess close to the anastomosis, even without any evident communication with the colonic lumen, was considered a leak. AL was graded (A–B–C) according to the intervention required,” according to the International Study Group of Rectal Cancer definition (15, 16). Neoplasms were classified according to the WHO classification (17) and staged according to the 8th Edition Staging System of the American Joint Committee on Cancer (AJCC) (18).

One day before colorectal surgery, bowel preparation (one package of Moviprep® (Norgine GmbH, Wettenberg, Germany) with 1 L of fluid orally) and selective oral bowel decontamination (standard solution with Colistin and Tobramycin, manufactured at our hospital pharmacy) was the standard of care for both sigmoid resection and total mesorectal excision (TME). Furthermore, 30–60 min before the operation, an intravenous single-shot antibiotic prophylaxis with cephalosporin of the 2nd or 3rd generation plus metronidazole was administered.

Patients with benign and malignant diseases were treated in the context of the national guidelines and according to international standard protocols (19–21). Technically, we follow the national guidelines by routinely performing a splenic flexure mobilization and a “high-tie” of the inferior mesenteric artery and inferior mesenteric vein in patients with colorectal cancer (20). TME was performed in a nerve-sparing manner along the so-called holy plane and visualizing the hypogastric plexus (22). TME was executed down to the pelvic floor to resect the whole mesorectum. Subsequently, the rectum was transected below the mesorectum with a linear stapler. The deep anastomosis was performed either as a transverse coloplasty or side-to-end in double stapling technique with a circular stapler of a minimum diameter of 28 mm. In the case of a sigmoid resection, either an end-to-end or a side-to-end anastomosis was performed. As a standard procedure, assessment of the integrity of the donuts and rectoscopy with an air leakage test was always performed (16). A diverting ileostomy was created in all patients with TME. In the case of TME, a flexible transrectal tube (20 charriere) was placed for 5 days.

### Indocyanine Green Near-Infrared Fluoroangiography

Intraoperatively, the oral bowel transection line in the descending colon was conventionally determined by a responsible surgeon under white light outside the abdominal cavity according to macroscopic perfusion. After marking this area with a suture, the bowel was consequently dissected in the macroscopically well-perfused region before the anvil was placed in the descending colon. After that, the anesthesiologist injected 5 ml (5 mg/ml) ICG dye (Pulson Medical Systems, Munich, Germany) intravenously, as per protocol. During injection, the descending colon was observed using the fluorescence mode of the PINPOINT endoscope Fluorescence Imaging System (Novadaq, Ontario, Canada), and angiography with the distribution of perfusion was assessed by a surgeon with a straight 0° telescope on a screen. As quantitative parameters were not available, the perfusion was assessed subjectively using a loop of the small intestine as a reference for perfusion by holding it along the region of transection at the large bowel for comparison (23). We defined a saturated green in the ICG-NIFA with visible small vessels at the transection line up to 90 s after dye injection as optimal vascularization. If there was no optimal perfusion, re-resection of the descending colon to the optimal level as indicated by ICG-NIFA was performed. In case of an optimal blood supply, anastomosis was performed. To ensure anastomotic integrity and macroperfusion, standard tests, such as circumferential visual control, rectoscopy with air leak test, and donut assessment for circular integrity after stapler anastomosis were performed. In case of questionable anastomotic perfusion, the ICG-NIFA assessment was repeated. Changes in the operative strategy (e.g., change of resection line, resection, and reconstruction of a new anastomosis) were documented in the operation report.

### Statistics

Data were presented as counts with percentages for categorical and as median with range for continuous variables. Categorical variables were compared by Pearson's chi-squared test or, in the case of expected frequencies <5, Fisher's exact test was used. To compare continuous variables with normal distribution, the *t*-test was used, while continuous variables without normal distribution were analyzed with the Mann–Whitney-*U*-test.

A univariate regression model was conducted to analyze the probability of occurrence to develop AL related to different risk factors. All variables with *p* < 0.15 were involved in a stepwise regression with forward selection. This method iteratively adds predictor variables from preset variables and tests their statistical significance, creating an incremental construction of a regression model. The process of choosing the variables is carried out automatically by the statistical software package. The basic criterion for this analysis was the occurrence of AL.

Additionally, several multivariate regression models were conducted and selected by the score criterion to examine models with up to six variables near to the highest score. This process revealed the stable trend and significance of the variables “no use of ICG-NIFA,” “previous abdominal surgery,” and “stage IV in case of cancer,” resulting in the inclusion of these factors in the final multivariate analysis. For all tests, *p* < 0.05 were defined as statistically significant.

Descriptive statistical analyses were performed using R Studio for Mac (Version 1.2.5042; RStudio Inc., Boston, MA, USA). Univariate and multivariate statistical analyses were conducted with SAS software (Version 9.4; SAS Institute Inc., Cary, NC, USA).

## Results

In the 3-year study period, 132 patients underwent elective sigmoid resection (*n* = 70) or TME (*n* = 62) with colorectal anastomosis. Among these, 70 (53%) were performed with ICG-NIFA and 62 (47%) without ICG-NIFA. Patients' clinical and intraoperative characteristics were similar in both groups (Table 1). Of the total, 45 patients were female and the median age at resection was 63.5 (range 33–93) years, and 100 patients were operated on for colorectal cancer and 32 patients for benign diseases. Of 60 patients with rectal cancer, 32 (53.3%) underwent neoadjuvant radiochemotherapy. A total of 66 patients (50%) had previous abdominal operations, 98 (74.2%) patients underwent laparoscopic/robotic resections and 34 (25.8%) conventional open resections. The procedures were performed by a total of 11 surgeons, including 2 surgeons certified as colorectal surgeons by the German Cancer Society. A total of 91 (69%) procedures were performed by certified colorectal surgeons, and 41 (31%) were supervised educational procedures. All patients underwent hydropneumatic testing of the anastomosis, and 8 patients (6.1%) showed intraoperative minor AL (2 sigmoid resections and 6 TME), which required additional suturing of the anastomosis to achieve airtightness.

**Table 1:** Patient demographic and clinical characteristics in the ICG-NIFA and no-ICG-NIFA groups.

**Variable**	**Total cohort** **(*n* = 132)**	**ICG-NIFA^a^** **(*n* = 70, 53%)**	**No-ICG-NIFA** **(*n* = 62, 47%)**	***p*-value**
Gender, *n* (%)				
Female	45 (34.1)	22 (31.4)	23 (37.1)	
Male	87 (65.9)	48 (68.6)	39 (62.9)	0.4929
Median age (years)	63.5 (33–93)	66.5 (34–88)	59.5 (33-93)	0.07262
Median BMI^b^ (kg/m^2^)	26.7 (14.9–44.8)	26.7 (19.6–36.1)	26.7 (14.9–44.8)	0.7278
<18.5	1 (0.8)	0 (0)	1 (1.6)	
18.5–30	100 (76.3)	55 (79.7)	45 (72.6)	
>30	30 (22.9)	14 (20.3)	16 (25.8)	0.4659
Diabetes mellitus, *n* (%)				
Yes	19 (14.4)	8 (11.4)	11 (17.7)	
No	113 (85.6)	62 (88.6)	51 (82.3)	0.3024
Treatment with anticoagulants or thrombocyte aggregation inhibitors, *n* (%)				
Yes	33 (25.0)	18 (25.7)	15 (24.2)	
No	99 (75.0)	52 (74.3)	47 (75.8)	0.8404
Corticosteroid treatment, *n* (%)				
Yes	3 (2.3)	3 (4.3)	0 (0)	
No	129 (97.7)	67 (95.7)	62 (100.0)	0.2471
Continuous alcohol intake *n* (%)				
Yes	13 (10.2)	7 (10.4)	6 (10.0)	
No	114 (89.8)	60 (89.6)	54 (90.0)	0.9338
Smoker, *n* (%)				
Yes	22 (17.3)	9 (13.4)	13 (21.7)	
No	105 (82.7)	58 (86.6)	47 (78.3)	0.2209
ASA^c^, *n* (%)				
1, 2	72 (54.5)	38 (54.3)	34 (54.8)	
3	59 (44.7)	32 (45.7)	27 (43.5)	
4	1 (0.8)	0 (0)	1 (1.6)	0.7869
Diagnosis, *n* (%)				
Cancer	100 (75.8)	57 (81.4)	43 (69.4)	
Benign pathology	32 (24.2)	13 (18.6)	19 (30.6)	0.1062
Stage, AJCC^d^ 8th, *n* (%)				
0/I	25 (24.8)	14 (24.6)	11 (25.0)	
II	31 (30.7)	16 (28.1)	15 (34.1)	
III	34 (33.7)	18 (31.6)	16 (36.4)	
IV	11 (10.9)	9 (15.8)	2 (4.5)	0.3567
Previous abdominal surgery, *n* (%)	66 (50.0)	35 (50.0)	31 (50.0)	1
Neoadjuvant RCTC^e^, *n* (%)	32 (53.3)	17 (53.1)	15 (53.6)	0.9724
Length of hospital stay (days)	10.5 (5–94)	10 (7–44)	11 (5–94)	0.1872

a *ICG-NIFA, Indocyanine green near-infrared fluoroangiography*.

b *BMI, Body Mass Index*.

c *ASA, American Society of Anaesthesiologists*.

d *AJCC, American Joint Committee on Cancer*.

e *RCTC, Radiochemotherapy, for rectal cancer only*.

**Figure 1:**
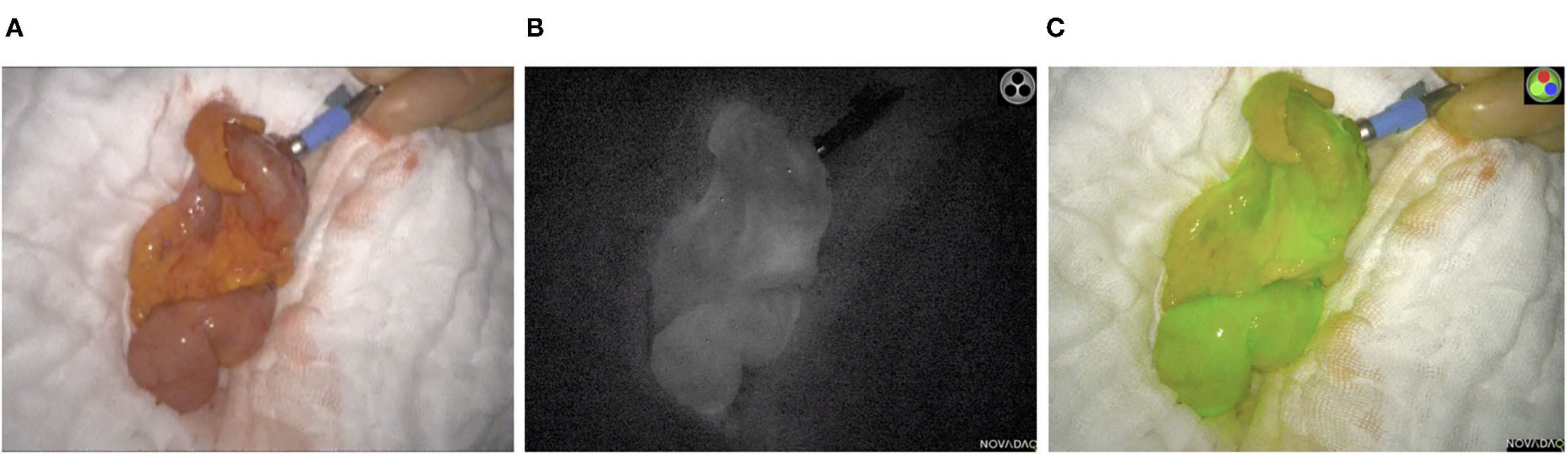
Intraoperative ICG-NIFA showing an optimal perfused descended colon after resection, 60 s after intravenous ICG application. **(A)** View with standard light. **(B)** View with NIR light. **(C)** View with the fluorescence mode of the PINPOINT endoscope Fluorescence Imaging System.

**Figure 2:**
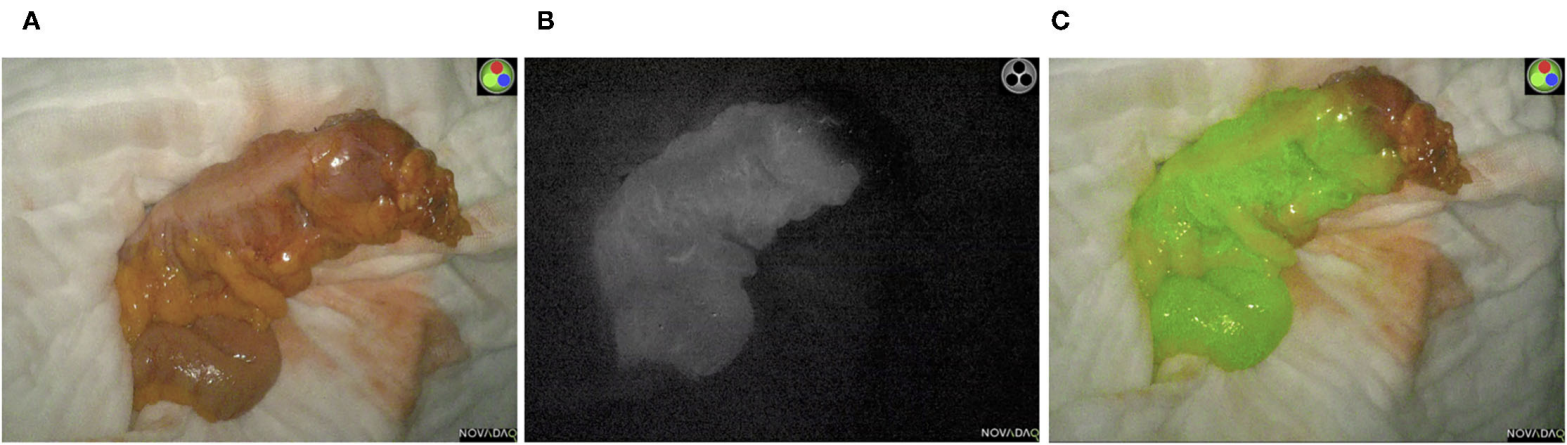
Intraoperative ICG-NIFA showing an inadequate perfused descended colon after dissection requiring re-resection. **(A)** View with standard light. **(B)** View with NIR light. **(C)** View with the fluorescence mode of the PINPOINT endoscope Fluorescence Imaging System.

In the ICG-NIFA group, no adverse events due to ICG administration were observed. In this group, visible fluorescence was seen in all 70 patients after almost 90 s (Figure 1). The median operative time was not significantly different between the ICG-NIFA and no-ICG-NIFA groups (sigmoid resection 182.5 vs. 189 min, *p* = 0.9577; TME 260 vs. 279 min, *p* = 0.9047). In 9 patients (12.9%), a re-resection of the descending colon was performed because blood microperfusion by ICG-NIFA was assessed as suboptimal (Figure 2). All anastomosis after bowel re-resection appeared well-perfused (Table 2), and none of the patients who underwent re-resection of the descending colon developed AL.

**Table 2:** Intra- and post-operative data in the ICG-NIFA and no-ICG-NIFA groups.

**Variable**	**Total cohort** **(*n* = 132)**	**ICG-NIFA^a^** **(*n* = 70)**	**no-ICG-NIFA** **(*n* = 62)**	***p*** **-value**
Intraoperative data				
Procedure, *n* (%)				
Sigmoid resection	70 (53.0)	38 (54.3)	32 (51.6)	
TME^b^	62 (47.0)	32 (45.7)	30 (48.4)	0.7588
Surgical approach, *n* (%)				
Conventional open	34 (25.8)	14 (20.0)	20 (32.3)	
Laparoscopic/robotic	98 (74.2)	56 (80.0)	42 (67.7)	0.108
Surgical expertise, *n* (%)				
Certified colorectal^c^ surgeon	91 (68.9)	48 (68.6)	43 (69.4)	
Educational procedure	41 (31.1)	22 (31.4)	19 (30.6)	0.9227
Median operative time (minutes)	220 (100–457)	222.5 (120–420)	214 (100–457)	0.9455
Anastomosis technique, *n* (%)				
Stapler	128 (97.0)	70 (100.0)	58 (93.5)	
Hand sewn	4 (3.0)	0 (0)	4 (6.5)	0.04617
Protective loop-ileostomy, *n* (%)	61 (46.2)	32 (45.7)	29 (46.8)	0.903
Intraoperative bowel re-resection, *n* (%)	14 (10.6)	9 (12.9)	5 (8.1)	0.3721
Intraoperative anastomotic leakage, *n* (%)	8 (6.1)	3 (4.3)	5 (8.1)	0.4737
Perioperative blood transfusion, *n* (%)	9 (6.8)	5 (7.1)	4 (6.5)	1
Post-operative data				
Anastomotic leakage (AL), *n* (%)	10 (7.6)	1 (1.4)	9 (14.5)	0.006166
Grade A	0 (0)	0 (0)	0 (0)	
Grade B	1 (0.8)	0 (0)	1 (1.6)	
Grade C	9 (6.8)	1 (1.4)	8 (12.9)	0.007459
AL in sigmoid resections, *n* (%)	4 (5.7)	0 (0)	4 (12.5)	0.03922
AL in TMEs, *n* (%)	6 (9.7)	1 (3.1)	5 (16.7)	0.09858
Reoperation because of AL with stoma creation, *n* (%)	3 (2.3)	0 (0)	3 (4.8)	0.1009
Overall abdominal morbidity, *n* (%)	23 (17.4)	8 (11.4)	15 (24.2)	0.05365
Re-op. for bowel obstruction	6 (4.5)	2 (2.9)	4 (6.5)	0.419
Re-op. for hemorrhage	2 (1.5)	1 (1.4)	1 (1.6)	1
Re-op. for abscess	0	0	0	
Re-op. for other causes	6 (4.5)	2 (2.9)	4 (6.5)	0.6838
Dindo-Clavien ≥3, *n* (%)	27 (20.5)	10 (14.3)	17 (27.4)	0.0619
Mortality, *n* (%)	0	0	0	

a *ICG-NIFA, Indocyanine green near-infrared fluoroangiography*.

b *TME, Total Mesorectal Excision*.

c *CRC, Colorectal Certified*.

Overall, 10 (7.6%) patients developed AL, which was significantly higher in the no-ICG-NIFA group (*n* = 9, 14.5%) compared to the ICG-NIFA group (*n* = 1, 1.4%; *p* = 0.006). In the group of sigmoid resections, 4 of the 32 (12.5%) patients without ICG-NIFA compared to 0 of 38 patients with ICG-NIFA developed AL (*p* = 0.04). In the TME group, 1 of 32 (3.1%) with ICG-NIFA compared to 5 of 30 (16.7%) without ICG-NIFA experienced AL (*p* = 0.0986). All four patients with sigmoid resections had type C AL, whereas in the TME group, two patients had type B and 4 patients had type C AL, respectively (Table 2).

Multivariate analysis of risk factors influencing AL demonstrated the use of ICG-NIFA (RR 34.926, 95%CI 2.106; 579.265, *p* = 0.0132) as an independent factor to reduce the AL rate (Table 3).

**Table 3:** Uni and multivariate analyses of risk factors for the development of post-operative AL^a^.

**Factor**	**Univariate analysis HR (95%CI) *p*-value**	**Multivariate analysis HR (95%CI) *p*-value**
Age ≥ 64 years	1.000 [0.275; 3.631] *p* = 1	
Male gender	2.177 [0.442; 10.712] *p* = 0.3386	
BMI^b^ ≥ 30	2.321 [0.610; 8.825] *p* = 0.2166	
Diabetes mellitus	0.642 [0.077; 5.379] *p* = 0.6828	
Treatment with anticoagulants or thrombocyte aggregation inhibitors	0.734 [0.148; 3.643] *p* = 0.7052	
Corticosteroid treatment	<0.001 [<0.001; >999.999] *p* = 0.9831	
Continuous alcohol intake	<0.001 [<0.001; >999.999] *p* = 0.9656	
Smoking	2.606 [0.599; 11.335] *p* = 0.2017	
ASA^c^ ≥3	3.038 [0.750; 12.307] *p* = 0.1196	
Diagnosis of cancer	0.695 [0.169; 2.866] *p* = 0.6148	
Stage IV (in case of cancer)	3.139 [0.578; 17.040] *p* = 0.1851	16.401 [1.165; 230.794] *p* = 0.0381
Neoadjuvant treatment (in TME^d^)	2.238 [0.590; 8.494] *p* = 0.2364	
Previous abdominal surgery	0.227 [0.046; 1.111] *p* = 0.0672	0.189 [0.034; 1.058] *p* = 0.0580
Educational procedure	2.389 [0.652; 8.759] *p* = 0.1889	
Open surgery	3.208 [0.867; 11.861] *p* = 0.0807	
Stapler anastomosis	0.227 [0.021; 2.409] *p* = 0.2185	
Perioperative blood transfusion	1.583 [0.178; 14.102] *p* = 0.6804	
No use of ICG-NIFA^e^	11.711 [1.439; 95.284] ***p*** **= 0.0214**	34.926 [2.106; 579.265] *p*=0.0132

a *AL, Anastomotic Leakage*.

b *BMI, Body Mass Index*.

c *ASA, American Society of Anaesthesiologists*.

d *TME, Total Mesorectal Excision*.

e *ICG-NIFA, Indocyanine green near-infrared fluoroangiography*.

*The meaning of the bold values is the significance of the factor*.

## Discussion

Anastomotic leakage requiring intervention (Grade B/C) is a severe primary complication after left-sided colorectal surgery, which leads to significant morbidity and hospital mortality of 5–7.5% in case of failure to be rescued (16, 24, 25). The overall rate of AL was 9.3% in German-certified clinics for colorectal cancer treatment according to a recent survey of 287,227 patients between 2012 and 2017 (25). The reported rates of AL after TME range between 3 and 21% (26, 27). The overall rate of AL in the present retrospective study lies within 7.5% below that level, and 9.7% AL rate after TME lies within the reported range.

Adequate perfusion of the anastomosed bowel ends is regarded as crucial for optimal anastomotic healing. Low tissue oxygenation based on inadequate perfusion was shown to play an essential role in the development of AL (28). In an ongoing effort to reduce the rate of AL in the setting of a teaching clinic, the ICG-NIFA was introduced in 2017, thereafter few small retrospective studies suggested that this method might be an accurate method for the intraoperative assessment of the anastomotic perfusion, potentially reducing the AL rate (7, 29). The still commonly used technique for evaluating local bowel perfusion at present is the subjective clinical estimation by the operating surgeon based on subjective criteria such as the color of serosa and mucosa, bleeding of the bowel ends, and pulsation of nearby small vessels. According to a recent analysis of the EURO-FIGS registry, in 27% of colorectal procedures, ICG-NIFA was used to assess colonic perfusion (30). In the present retrospective unicentric study in the setting of a teaching clinic, the overall AL rate after left colectomy (sigmoid resections and TMEs) of 1.4% was significantly lower in the ICG-NIFA group compared to 14.5% in the no-ICG-NIFA group (*p* = 0.006). The AL rate after TME was also lower in the ICG-NIFA-group in comparison with the group without the use of ICG-NIFA (3.1 vs. 16.7%, *p* = 0.0986). This is in line with a recent meta-analysis of retrospective trials that reported a reduction of colorectal AL rates using ICG-NIFA from 9.2 to 3.3% (10). Moreover, the current findings correspond to the results of other extensive studies. A previous multicenter clinical trial with 139 patients with both benign and malignant diseases evaluated the perfusion assessment during left-sided colectomy and anterior resection with the result of a lower AL rate (1.4%) in comparison with reported rates in the literature. However, this was a single-armed study of moderate size with a heterogeneous population and a non-standardized perioperative procedure. Therefore, a definite conclusion cannot be drawn (11). In addition, the results were consistent with a multicenter phase II trial including 504 patients with benign and malignant pathologies undergoing any colorectal resections, where the AL rate was 2.4% in the ICG-NIFA group compared to a rate of 5.8% obtained from historical operation data from the same surgeons without ICG-NIFA. In particular for left-sided operations, there was a significant difference between the ICG-NIFA and the historical control group (2.6 vs. 6.9%, *p* = 0.005). Nevertheless, the power of the study was also limited by its non-randomized character (12).

A recent Italian prospective randomized controlled multicentric trial randomized 252 patients undergoing left-sided colorectal resections to either ICG-NIFA or subjective visual evaluation of the bowel perfusion without ICG; however, it revealed no significant reduction of the AL rate in the ICG-NIFA arm (5 vs. 9%, *p* >0.05) (31). Another very recent randomized controlled multicenter study confirmed these results, focusing on low anterior resections procedures including 347 patients with rectal cancer (9.0% in the ICG-NIFA group vs. 9.6% in standard evaluation, *p* > 0.05). Also, the multivariate analysis revealed no significant difference between the ICG-NIFA and the standard group. Nevertheless, it has to be stated that the statistical power of the trial was limited because the predetermined sample size could not be achieved (13).

The subject of several studies was the change of the colonic resection line after the intraoperative use of ICG-NIFA. In the literature, a strategical change of the resection line was reported between 2.5 and 25% of colorectal resections and about 12–20% of left-sided resections (8, 31). This was the case in 12.9% (*n* = 9) of the patients in the present study. Although a change of the colonic resection line can be regarded as the major impact of ICG-NIFA, the definitive proof of a correlation between the use of ICG-NIFA and reduction of the AL rate is still missing. If optimal perfusion assessed by ICG-NIFA would be the only factor influencing anastomotic healing, no AL should occur.

Nevertheless, the etiology of AL depends on many factors. Male sex, high ASA score ≥III, cigarette smoking, alcohol consumption, perioperative blood transfusion, no protective ileostomy, higher UICC stages, low rectal cancer, and neoadjuvant radiochemotherapy were reported to be independent risk factors for AL (16, 32, 33). Also, it is known that conducting the resection in an acute phase of inflammation, for example, in the case of acute diverticulitis, is associated with a higher risk of developing AL. Due to difficult conditions, further actions to avoid complications can be required (34). However, this factor did not play a role in the present study since emergency procedures were excluded. Overall, patient groups in the present study contained all different risk factors, but the patients' clinical characteristics in the ICG-NIFA and no-ICG-NIFA groups were homogenous and comparable for statistical evaluation. The use of ICG-NIFA in the present cohort was the only independent positive prognostic factor to reduce AL. However, it has to be stated that the sample size was relatively underpowered to arrive at a definitive conclusion.

The technique of ICG-NIFA in the present study was different from most previous studies, which primarily performed the ICG-NIFA before bowel transection. In the present study, the ICG-NIFA was first performed after transection of the descending colon and incorporating the stapler anvil. Since this was always performed before the distal sigmoid or rectal dissection, there was a 15–60 min delay before the ICG-NIFA could be performed. If the ICG-NIFA assessment of the blood supply was regarded as optimal, the perfusion remained most likely stable.

The present study is limited by its retrospective nature and the use of the ICG-NIFA by choice. Therefore, better outcomes after using ICG-NIFA may also be attributable to the improvement of surgeons, improved capability over time, or improvement of patients' management, rather than the use of ICG-NIFA. Moreover, the use of ICG-NIFA could have produced better outcomes due to an unconscious modification of surgical behavior in response to the awareness of being under observation for surgical outcomes (35).

Also, the adequacy of anastomotic perfusion following ICG-NIFA was still assessed subjectively by a surgeon since no validated objective analytic method is currently available. Especially lacking quantification of the intensity and the fact that the perfusion assessment may be dependent on the camera and the distance from the light source and the target remains a major limitation of ICG-NIFA. Moreover, there are no concrete standards concerning the ideal injection dose and the optimal time to evaluate the perfusion after injection (36). Furthermore, the basic concept of ICG-NIFA currently does not include the diffusion of fluorescence molecules over time. This predominantly static technique may lead to overestimating border zones of ischemic areas due to the capillary flow diffusion of the dye over time. Therefore, it is necessary to evaluate this technique to achieve a standardized, replicable assessment (37).

In conclusion, ICG-NIFA is an easy-to-use straightforward technique that allows the assessment of the large bowel perfusion without adverse effects. In the setting of a teaching clinic, its non-randomized use was associated with a significant reduction in the post-operative AL rate. Based on these results, ICG-NIFA was established as the standard procedure for the assessment of bowel perfusion and determination of the resection line for left-sided colorectal resections in our clinical practice. In the future, real-time analysis combined with artificial intelligence providing objective perfusion parameters might even lead to better safety for the patients.

## Data Availability Statement

The raw data supporting the conclusions of this article will be made available by the authors, without undue reservation.

## Ethics Statement

The studies involving human participants were reviewed and approved by Ethics Committee of the Philipps-University Hospital, Marburg, Germany. Written informed consent for participation was not required for this study in accordance with the national legislation and the institutional requirements.

## Author Contributions

DB contributed to conception and design of the study. MN organized the database, analyzed the data, performed the statistical analysis, and wrote the manuscript. DB, EM, and VK enrolled patients, checked the data analysis, revised the manuscript. All authors contributed to the article and approved the submitted version.

## Conflict of Interest

The authors declare that the research was conducted in the absence of any commercial or financial relationships that could be construed as a potential conflict of interest.

## Publisher's Note

All claims expressed in this article are solely those of the authors and do not necessarily represent those of their affiliated organizations, or those of the publisher, the editors and the reviewers. Any product that may be evaluated in this article, or claim that may be made by its manufacturer, is not guaranteed or endorsed by the publisher.
